# Editorial: Perinatology in the Era of Big Data and Nanoparticles

**DOI:** 10.3389/fped.2015.00095

**Published:** 2015-11-05

**Authors:** Martin G. Frasch

**Affiliations:** ^1^Department of Obstetrics and Gynecology, CHU Sainte-Justine Centre de Recherche, Université de Montréal, Montreal, QC, Canada; ^2^Department of Neurosciences, CHU Sainte-Justine Centre de Recherche, Université de Montréal, Montreal, QC, Canada

**Keywords:** monitoring, labor, microbiome, fetus, neonate, big data integration, electronic medical record, perinatal care

How do prenatal exposures to various stimuli impact postnatal development for the duration of a person’s life? To answer this, the tripartite challenge is biological, medical, and technological. The biological challenge is to understand the plethora of effects causing trajectory shifts. The medical and technological challenges are to identify and follow fetuses and babies at risk for diseases in later life. Systems to accomplish this must be deployable across variably equipped healthcare settings at a reasonable cost.

Many researchers believe that such challenges can be tackled with complex signals bioinformatics. This research topic attracted articles on fetal heart rate (FHR) monitoring during labor, optimization of technologies for pediatric ventilation and the impact of the developing neonatal microbiome on health over the life span. How do these topics connect? They all share a clinical and translational demand for integrating relatively large amounts of spatio-temporally distributed data from various modalities to reveal patterns not clearly discernible to the human eye, with the goal of optimizing medical decision-making.

The right idea has to arrive at the right time to be met by a technology that can implement it, to be taken up by practitioners, and to produce change in health outcomes (1–3). Although technology seems at hand, a paradigm shift is required for practitioners to embrace this challenge. We can no longer afford to rely on human perception alone to detect patterns in the twenty-first century’s onslaught of multi-modal and multi-dimensional data streams reflecting human health in acute and chronic care settings (4). Such data streams are commonly referred as Big Data.

Big data can be defined with three Vs: “*high-volume, high-velocity, and high-variety information assets that demand cost-effective, innovative forms of information processing for enhanced insight and decision making*” (5).

In this research topic, Durosier et al. show that sampling rate affects the ability of FHR monitoring to detect acidemia as it occurs during human labor (6). Acquiring ~250 times more data points per second than currently practiced in delivery rooms worldwide improves accuracy. New technologies, some re-discovered form the early 1980s, are now coming to market to address this need, although their indication for use in delivery rooms is not yet fully exploiting their potential (7–11). As current computing capacity no longer limits the sampling rate possibilities for online monitoring, “bigger” data are becoming a logical next step in improving health care (12). One challenge is the integration of live streams into electronic medical records to facilitate retrieval for diagnostic and research purposes and for medical decision-making in real time.

The common thread connecting Durosier’s work to the next study in the research topic is the notion of unveiling physiological variability using higher temporal resolution of data acquisition and modalities of human–machine interaction that account for the natural biological variability. In the case of fetal monitoring, this variability is contained in the subtle temporal FHR fluctuations that remain hidden to human eye when acquired with the conventional tools at a 4-Hz sampling rate.

Baudin et al. explore how biological variability is impacted by the various mechanical ventilation regimes when it comes to monitoring breathing patterns in infants requiring machine support (13, 14). Intuitively, machine ventilation algorithms that are most closely attuned to the physiological respiratory pattern produce breathing signals that most closely resemble those of the control infants. Although larger prospective studies are necessary to understand the differential impact of ventilatory modes on cardio-respiratory variability and their effect on clinical outcomes, this study shows the possibility of deploying off- or online tools to quantify the physiological variability in respiration from bedside pediatric data streams. In the long term, this might help to fine-tune the ventilation parameters beyond the current possibilities, accounting for the non-linear nature of respiratory patterns. Again, the theme of how biological variability can be usefully monitored in real time emerges.

Munyaka et al. explore early postnatal maturation of immune regulation as a function of the exposure to gut microbiome (15). The human gut houses up to 10^14^ bacteria, exceeding by ~10-fold the number of host cells. Microbiome–host immune system interactions appear to have a profound and life-lasting impact on the host’s health status beginning well before the baby is born (16–21). We are faced with the challenge of quantifying microbial diversity in space and time, which approximates at least two of the above “3 Vs” definition of the big data, variety, and volume. Although the previous manuscripts represent intuitive approaches to temporal profiling and pattern analysis of streaming data, embodying all three Vs, current approaches to microbiome analysis need to catch up to the third V (velocity) to provide higher spatiotemporal resolution (microbiome in different organs at various time points). Meanwhile, current studies are cross-sectional in nature, sometimes with multiple sampling of the same cohort over time. They can offer population-level insights into changes in the microbiome due to various exposures, but with low temporal or spatial resolution to gage intra- and interindividual microbial dynamics.

Sorani et al. provided an early proof-of-principle for creation of multivariate pattern recognition within physiological time series commonly acquired in an intensive care unit setting (22). Heat maps in which genes are displayed across the top row and related genes cluster together are commonly used in genetics. In their neurocritical care heat map, Sorani et al. replaced genes by physiological variables that cluster on the basis of association within and across patients into three groups of patients. Surprisingly, intracranial pressure (ICP) and fraction of inspired oxygen were clustered, leading to the identification of previously unrecognized ICP elevations during bedside suctioning. As a perinatal example of unexpected connections, multi-dimensional properties of fHRV encode signatures of inflammation (23–26) or progressive labor acidemia (10, 11, 27) and may relate to EEG parameters (28, 29). Modern machine learning will help to integrate microbiome indices and continuous bedside acquisition of multi-modal data to elucidate clinically relevant patterns and optimize treatment (4, 22). Data intelligence is the next logical step in evolution of health monitoring (2, 3, 30).

Focusing machine learning approaches on clinical questions that arise in perinatal medicine is needed as relatively little progress has been made in the past decades in regard to monitoring and translation into clinical practice, with the notable exception of the HeRo score monitors (31, 32). Recent advances in artificial intelligence have brought the vision of data-driven case identification and decision-making closer to reality (33–37).

## Conflict of Interest Statement

Martin G. Frasch is a co-inventor of the related patent application entitled “EEG Monitor of Fetal Health” including U.S. Patent Application Serial No. 12/532,874 and CA 2681926 National Stage Entries of PCT/CA08/00580 filed March 28, 2008, with priority to US provisional patent application 60/908,587, filed March 28, 2007. No other conflicts of interest are declared.
